# Supplementary material for: Modeling glioblastoma heterogeneity as a dynamic network of cell states
Source: Mol Syst Biol. 2021 Sep 16;17(9):e10105. doi: 10.15252/msb.202010105 (PMC8444284; doi:10.15252/msb.202010105)
Supplement: Supplementary file 5 — Source Data for Figure 3 [file MSB-17-e10105-s001.zip › Figure3A_sourcedata/GSEA_3065/hallmarks_state6.GseaPreranked.1623416308702/HALLMARK_PI3K_AKT_MTOR_SIGNALING.html]

Details for gene set HALLMARK\_PI3K\_AKT\_MTOR\_SIGNALING[GSEA]

|  || Dataset | state6 |
| Phenotype | NoPhenotypeAvailable |
| Upregulated in class | na\_pos |
| GeneSet | HALLMARK\_PI3K\_AKT\_MTOR\_SIGNALING |
| Enrichment Score (ES) | 0.24074394 |
| Normalized Enrichment Score (NES) | 0.9888291 |
| Nominal p-value | 0.48022598 |
| FDR q-value | 0.5832857 |
| FWER p-Value | 1.0 |
Table: GSEA Results Summary

  

Fig 1: Enrichment plot: HALLMARK\_PI3K\_AKT\_MTOR\_SIGNALING      
 Profile of the Running ES Score & Positions of GeneSet Members on the Rank Ordered List

  

| PROBE | GENE SYMBOL | GENE\_TITLE | RANK IN GENE LIST | RANK METRIC SCORE | RUNNING ES | CORE ENRICHMENT || 1 | CDKN1A |  |  | 1 | 0.477 | 0.0836 | Yes |
| 2 | DDIT3 |  |  | 205 | 0.175 | 0.0911 | Yes |
| 3 | HRAS |  |  | 245 | 0.163 | 0.1153 | Yes |
| 4 | CAB39 |  |  | 570 | 0.116 | 0.0986 | Yes |
| 5 | MAP3K7 |  |  | 694 | 0.106 | 0.1031 | Yes |
| 6 | SQSTM1 |  |  | 719 | 0.104 | 0.1187 | Yes |
| 7 | PTPN11 |  |  | 804 | 0.098 | 0.1262 | Yes |
| 8 | RAC1 |  |  | 816 | 0.097 | 0.1420 | Yes |
| 9 | ACTR2 |  |  | 833 | 0.096 | 0.1570 | Yes |
| 10 | MYD88 |  |  | 915 | 0.090 | 0.1636 | Yes |
| 11 | UBE2N |  |  | 947 | 0.089 | 0.1756 | Yes |
| 12 | IRAK4 |  |  | 1091 | 0.081 | 0.1735 | Yes |
| 13 | NFKBIB |  |  | 1179 | 0.076 | 0.1769 | Yes |
| 14 | ADCY2 |  |  | 1298 | 0.071 | 0.1759 | Yes |
| 15 | PIK3R3 |  |  | 1321 | 0.070 | 0.1858 | Yes |
| 16 | GSK3B |  |  | 1382 | 0.068 | 0.1908 | Yes |
| 17 | AP2M1 |  |  | 1422 | 0.067 | 0.1981 | Yes |
| 18 | CDK4 |  |  | 1490 | 0.064 | 0.2018 | Yes |
| 19 | DUSP3 |  |  | 1618 | 0.060 | 0.1977 | Yes |
| 20 | ACTR3 |  |  | 1668 | 0.058 | 0.2023 | Yes |
| 21 | ATF1 |  |  | 1729 | 0.056 | 0.2052 | Yes |
| 22 | PTEN |  |  | 1739 | 0.055 | 0.2138 | Yes |
| 23 | AKT1S1 |  |  | 1750 | 0.055 | 0.2223 | Yes |
| 24 | PRKAR2A |  |  | 1839 | 0.052 | 0.2214 | Yes |
| 25 | RAF1 |  |  | 1924 | 0.050 | 0.2206 | Yes |
| 26 | ARF1 |  |  | 2015 | 0.047 | 0.2185 | Yes |
| 27 | GRK2 |  |  | 2026 | 0.047 | 0.2257 | Yes |
| 28 | E2F1 |  |  | 2034 | 0.047 | 0.2331 | Yes |
| 29 | MAPKAP1 |  |  | 2040 | 0.047 | 0.2407 | Yes |
| 30 | RALB |  |  | 2193 | 0.042 | 0.2307 | No |
| 31 | PIN1 |  |  | 2195 | 0.042 | 0.2379 | No |
| 32 | CFL1 |  |  | 2433 | 0.035 | 0.2169 | No |
| 33 | RIT1 |  |  | 2457 | 0.035 | 0.2203 | No |
| 34 | YWHAB |  |  | 2493 | 0.033 | 0.2222 | No |
| 35 | ECSIT |  |  | 2597 | 0.031 | 0.2158 | No |
| 36 | MAPK9 |  |  | 2835 | 0.026 | 0.1931 | No |
| 37 | TBK1 |  |  | 2840 | 0.026 | 0.1972 | No |
| 38 | MAPK8 |  |  | 2868 | 0.025 | 0.1985 | No |
| 39 | UBE2D3 |  |  | 3038 | 0.021 | 0.1828 | No |
| 40 | TNFRSF1A |  |  | 3049 | 0.021 | 0.1853 | No |
| 41 | ARHGDIA |  |  | 3321 | 0.015 | 0.1569 | No |
| 42 | TSC2 |  |  | 3324 | 0.015 | 0.1593 | No |
| 43 | RIPK1 |  |  | 3384 | 0.013 | 0.1549 | No |
| 44 | MAPK1 |  |  | 3810 | 0.004 | 0.1069 | No |
| 45 | MKNK2 |  |  | 3866 | 0.003 | 0.1012 | No |
| 46 | PRKAG1 |  |  | 4008 | 0.001 | 0.0851 | No |
| 47 | RPS6KA3 |  |  | 4022 | 0.000 | 0.0837 | No |
| 48 | STAT2 |  |  | 4078 | -0.001 | 0.0775 | No |
| 49 | ACACA |  |  | 4189 | -0.003 | 0.0654 | No |
| 50 | TIAM1 |  |  | 4472 | -0.008 | 0.0345 | No |
| 51 | CDKN1B |  |  | 4475 | -0.008 | 0.0357 | No |
| 52 | PLA2G12A |  |  | 4973 | -0.018 | -0.0182 | No |
| 53 | CSNK2B |  |  | 5064 | -0.020 | -0.0250 | No |
| 54 | AKT1 |  |  | 5071 | -0.020 | -0.0223 | No |
| 55 | EIF4E |  |  | 5088 | -0.020 | -0.0206 | No |
| 56 | NCK1 |  |  | 5409 | -0.027 | -0.0526 | No |
| 57 | PLCG1 |  |  | 5473 | -0.028 | -0.0549 | No |
| 58 | PPP2R1B |  |  | 6004 | -0.038 | -0.1090 | No |
| 59 | ARPC3 |  |  | 6141 | -0.041 | -0.1174 | No |
| 60 | SMAD2 |  |  | 6258 | -0.044 | -0.1230 | No |
| 61 | PPP1CA |  |  | 6444 | -0.048 | -0.1357 | No |
| 62 | MAPK10 |  |  | 6491 | -0.049 | -0.1323 | No |
| 63 | PAK4 |  |  | 6638 | -0.054 | -0.1395 | No |
| 64 | PFN1 |  |  | 6817 | -0.059 | -0.1496 | No |
| 65 | MAP2K3 |  |  | 6986 | -0.064 | -0.1575 | No |
| 66 | ITPR2 |  |  | 7079 | -0.067 | -0.1563 | No |
| 67 | CXCR4 |  |  | 7242 | -0.072 | -0.1621 | No |
| 68 | PIKFYVE |  |  | 7612 | -0.087 | -0.1892 | No |
| 69 | PRKAA2 |  |  | 7662 | -0.089 | -0.1792 | No |
| 70 | GRB2 |  |  | 7882 | -0.100 | -0.1868 | No |
| 71 | CDK2 |  |  | 8018 | -0.108 | -0.1832 | No |
| 72 | VAV3 |  |  | 8058 | -0.111 | -0.1682 | No |
| 73 | EGFR |  |  | 8251 | -0.126 | -0.1681 | No |
| 74 | CDK1 |  |  | 8351 | -0.139 | -0.1550 | No |
| 75 | SLC2A1 |  |  | 8552 | -0.173 | -0.1475 | No |
| 76 | CLTC |  |  | 8574 | -0.179 | -0.1183 | No |
| 77 | CALR |  |  | 8773 | -0.330 | -0.0830 | No |
| 78 | HSP90B1 |  |  | 8794 | -0.487 | 0.0002 | No |
Table: GSEA details [plain text format]

  

Fig 2: HALLMARK\_PI3K\_AKT\_MTOR\_SIGNALING: Random ES distribution      
 Gene set null distribution of ES for **HALLMARK\_PI3K\_AKT\_MTOR\_SIGNALING**
